# Retroaortic Left Renal Vein: A Case Series and Clinical Significance in Renal Surgery

**DOI:** 10.7759/cureus.97168

**Published:** 2025-11-18

**Authors:** Andreas Koumenis, Dimosthenis Chrysikos, Vasileios Protogerou, Theodoros Mariolis-Sapsakos, Theodore Troupis

**Affiliations:** 1 Department of Anatomy, Medical School, National and Kapodistrian University of Athens, Athens, GRC; 2 Laboratory of Anatomy, Department of Nursing, National and Kapodistrian University of Athens, Athens, GRC

**Keywords:** anatomical variation, posterior nutcracker syndrome, renal surgery, retroaortic left renal vein, venous anomaly

## Abstract

The retroaortic left renal vein (RLRV) is a rare anatomical variant in which the left renal vein passes posterior to the aorta. Although often asymptomatic, awareness of this anomaly is essential for avoiding vascular injury during retroperitoneal or renal surgery. We report three incidental cases of RLRV identified during imaging studies performed for unrelated renal or urinary conditions.

A 53-year-old man presented with colicky left flank pain and multiple left renal calculi on computed tomography (CT). An incidental type I RLRV with orthotopic drainage into the inferior vena cava (IVC) at the L1-L2 level was detected. He opted for conservative management with elective laser lithotripsy planned. An 86-year-old woman presented with fever and bilateral flank pain due to urinary tract infection and left ureteric obstruction. Contrast-enhanced CT demonstrated bilateral pyelonephritis, left hydronephrosis, and a type II RLRV draining at the level of L4. She underwent ureteral stenting and intravenous antibiotics, with complete recovery. A 67-year-old woman presented with right flank pain due to a distal ureteric calculus. Non-contrast CT revealed a type III RLRV encircling the aorta. She was treated with double J stenting and conservative management, with spontaneous stone expulsion after one month. All patients were managed for their primary conditions, and the venous anomaly required no intervention. Although frequently incidental, RLRV has important surgical implications. Preoperative identification through contrast-enhanced imaging allows appropriate surgical planning and prevention of intraoperative vascular complications.

## Introduction

The retroaortic left renal vein (RLRV) is a rare anatomical variant in which the left renal vein courses posterior to the abdominal aorta before draining into the inferior vena cava (IVC) [1]. Normally, the left renal vein traverses anterior to the aorta at the level of the L1-L2 vertebrae, receiving tributaries from the adrenal, gonadal, and lumbar veins. During embryologic development, this pattern arises from the persistence of the ventral (preaortic) limb and regression of the dorsal (retroaortic) limb of the renal venous collar. In RLRV, this process is reversed, with persistence of the dorsal limb and regression of the ventral one, resulting in a venous pathway located behind the aorta [1].

Although often an incidental finding, the prevalence of RLRV ranges from 0.5% to 3.5% in autopsy studies and up to 9% in imaging-based studies using multidetector computed tomography (CT) or magnetic resonance angiography [2]. The increasing use of high-resolution cross-sectional imaging has led to more frequent recognition of such variants. Awareness of RLRV is clinically significant because its unrecognized presence can result in serious intraoperative complications, particularly during renal, retroperitoneal, or vascular procedures [3]. Injury to a retroaortic renal vein can cause uncontrollable hemorrhage, necessitating open conversion or leading to significant morbidity.

In addition to its surgical importance, RLRV has been implicated in the pathogenesis of posterior nutcracker syndrome (PNCS), where compression of the vein between the aorta and the vertebral column leads to left renal venous hypertension [4]. This may manifest as flank pain, hematuria, or gonadal varices, although most cases remain asymptomatic [4].

We report three incidental cases of RLRV identified during imaging for unrelated renal or urinary conditions. Each case illustrates a distinct anatomical subtype of RLRV. Through this series, we aim to highlight the embryologic basis, classification, and clinical relevance of this venous anomaly and to underscore the importance of preoperative imaging awareness in preventing intraoperative vascular injury during renal and retroperitoneal surgery.

## Case presentation

Case 1

A 53-year-old man presented to the emergency department with sudden-onset, colicky left flank pain radiating to the groin, associated with mild nausea but no vomiting or fever. His medical history included hypertension and hyperlipidemia, both well controlled on oral medications. He denied prior renal surgery or urinary tract infection. On examination, he was afebrile (36.8°C), with a blood pressure of 142/88 mmHg, pulse rate of 84 bpm, and SpO₂ of 99% on 21% FiO2. Abdominal examination revealed localized tenderness over the left costovertebral angle (CVA) and mild left flank discomfort without guarding or rigidity. No palpable masses were noted, and bowel sounds were normal. Laboratory investigations showed microhematuria and a C-reactive protein (CRP) of 40.2 mg/L (normal range (NR): <5 mg/L), with a slightly elevated creatine level of 1.47 mg/dL (NR: 0.7-1.3 mg/dL).

**Figure 1:**
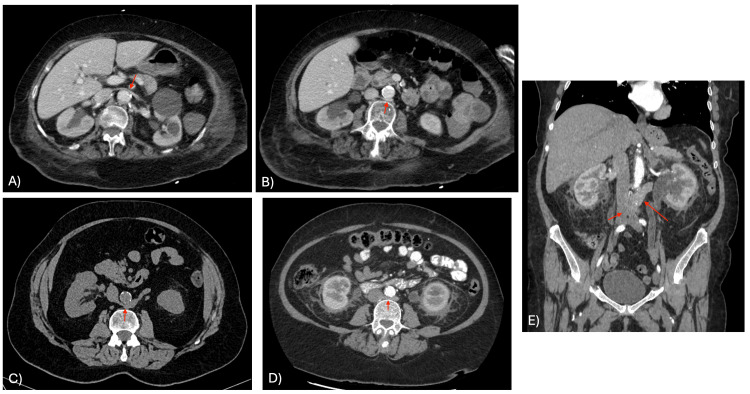
Type I, II, and III RLRV (A) Case 3: Contrast-enhanced CT demonstrating a circumaortic (type III) RLRV; the arrow points to the superior preaortic renal vein. (B) Case 3: Contrast-enhanced CT demonstrating the same type III RLRV, with the arrow indicating the retroaortic left renal vein. (C) Case 1: Axial non-contrast CT showing a type I RLRV (arrow) coursing posterior to the aorta and draining into the IVC at the L1-L2 level. (D) Case 2: Type II RLRV (arrow) draining into the IVC at L4. (E) Case 2: Coronal CT view demonstrating the type II RLRV; the short arrow indicates the entry point of the left renal vein into the IVC at L4, while the long arrow shows its retroaortic course. RLRV: retroaortic left renal veins, CT: computed tomography, IVC: inferior vena cava

A non-contrast CT scan demonstrated multiple left renal calculi without hydronephrosis. Incidentally, a type I RLRV was observed coursing posterior to the aorta and draining orthotopically into the IVC at the L1-L2 level (Figure 1C). The patient was counseled regarding treatment options and chose initial conservative management, with planned elective laser lithotripsy. The venous anomaly required no specific intervention.

Case 2

An 86-year-old woman with a history of type 2 diabetes mellitus and hypertension presented with fever, chills, and bilateral flank pain for four days. She denied hematuria or urinary retention. On arrival, she was febrile (38.4°C), tachycardic (102 bpm), and normotensive (128/72 mmHg), with a respiratory rate of 20 breaths per minute and oxygen saturation of 97% on room air. She appeared mildly dehydrated but alert. Physical examination revealed bilateral CVA tenderness, more pronounced on the left, with mild suprapubic discomfort. No palpable mass or peritonism was present. Urinalysis demonstrated hematuria, pyuria, and bacteriuria, consistent with urinary tract infection. Serum creatinine was elevated at 1.36 mg/dL (NR: 0.7-1.1 mg/dL), white blood cell (WBC) was 16.300/μL (NR: 4,500-11,000/μL), and CRP was 130 mg/L (NR: <5 mg/L).

A contrast-enhanced CT scan was performed to evaluate for renal abscess, which revealed bilateral pyelonephritis, left hydronephrosis, and a distal ureteric calculus at the vesicoureteric junction. Incidentally, a type II RLRV was identified, coursing posterior to the aorta and draining into the IVC at the L4 vertebral level (Figure 1D, 1E). She underwent cystoscopic double J ureteral stenting and received intravenous antibiotics. Her fever and pain resolved within five days, and repeat imaging confirmed resolution of infection and hydronephrosis. The RLRV was incidental and did not require intervention.

Case 3

A 67-year-old woman with a medical history of hypertension, dyslipidemia, and prior cholecystectomy presented with acute right flank pain and dysuria of two days’ duration. She denied hematuria, fever, or vomiting. On examination, her vital signs were stable (temperature: 37.1°C, pulse: 78 bpm, blood pressure: 136/80 mmHg, SpO₂: 98% on room air). She appeared in mild distress due to pain. Localized tenderness was elicited over the right CVA and right lower flank, without guarding or palpable mass. No abdominal anomaly was palpable. Laboratory tests revealed leukocytosis (15,170/μL; NR: 4,500-11,000/μL), serum creatinine of 1.47 mg/dL (NR: 0.7-1.1 mg/dL), and microhematuria on urinalysis.

Renal ultrasonography showed right hydronephrosis, and a CT scan confirmed a distal right ureteric calculus consistent with obstructive urolithiasis. Incidentally, a type III RLRV (circumaortic venous ring) was visualized, with preaortic and retroaortic limbs encircling the aorta before joining the IVC (Figure 1A, 1B). The patient underwent cystoscopic placement of a double J ureteral stent and was managed conservatively with antibiotics, hydration, and analgesia. Follow-up CT imaging one month later demonstrated complete stone passage and resolution of hydronephrosis. The venous anomaly was asymptomatic and required no treatment.

Informed oral consent was obtained from all patients for publication of this report and accompanying images.

## Discussion

The development of IVC and its tributaries begins between the sixth and eighth weeks of gestation [5]. This process involves the sequential appearance, regression, and anastomosis of three paired venous systems: the posterior cardinal, subcardinal, and supracardinal veins. The final renal venous anatomy arises from a vascular network known as the aortic collar, which forms around the abdominal aorta and connects the precursors of the IVC. The aortic collar consists of ventral (anterior) and dorsal (posterior) arches [5]. Under normal circumstances, the dorsal arch regresses, while the ventral arch persists, forming the normal left renal vein that courses anterior to the aorta [6].

The pathogenesis of an RLRV occurs when this sequence is reversed or incomplete. The ventral, preaortic arch regresses, while the dorsal, retroaortic arch persists, becoming the main venous conduit for left renal drainage posterior to the aorta [6]. The RLRV represents one spectrum within a continuum of anomalies derived from the same embryologic framework. It shares a common origin with the circumaortic left renal vein, which results from the persistence of both the ventral and dorsal arches of the aortic collar [6].

RLRV formation is not always confined to the aortic collar. Coexisting IVC malformations, such as agenesis of the renal or suprarenal segments, suggest a broader failure in the timing or pattern of consolidation within the subcardinal-supracardinal plexus during the critical sixth to eighth week [7]. When such developmental abnormalities occur, compensatory collateral pathways, including the azygos and hemiazygos veins, may become markedly dilated and crucial for systemic venous return. Recognizing these collaterals on preoperative imaging is essential, as they are highly susceptible to injury during upper pole tumor dissection.

The anatomical configuration of RLRV varies according to its termination. A caudally coursing RLRV that drains into the left common iliac vein represents the most extreme variant [8]. This configuration suggests failure of the dorsal arch to join the central IVC precursor and, potentially, incomplete formation of the infrarenal IVC, forcing a more caudal drainage site.

**Figure 2:**
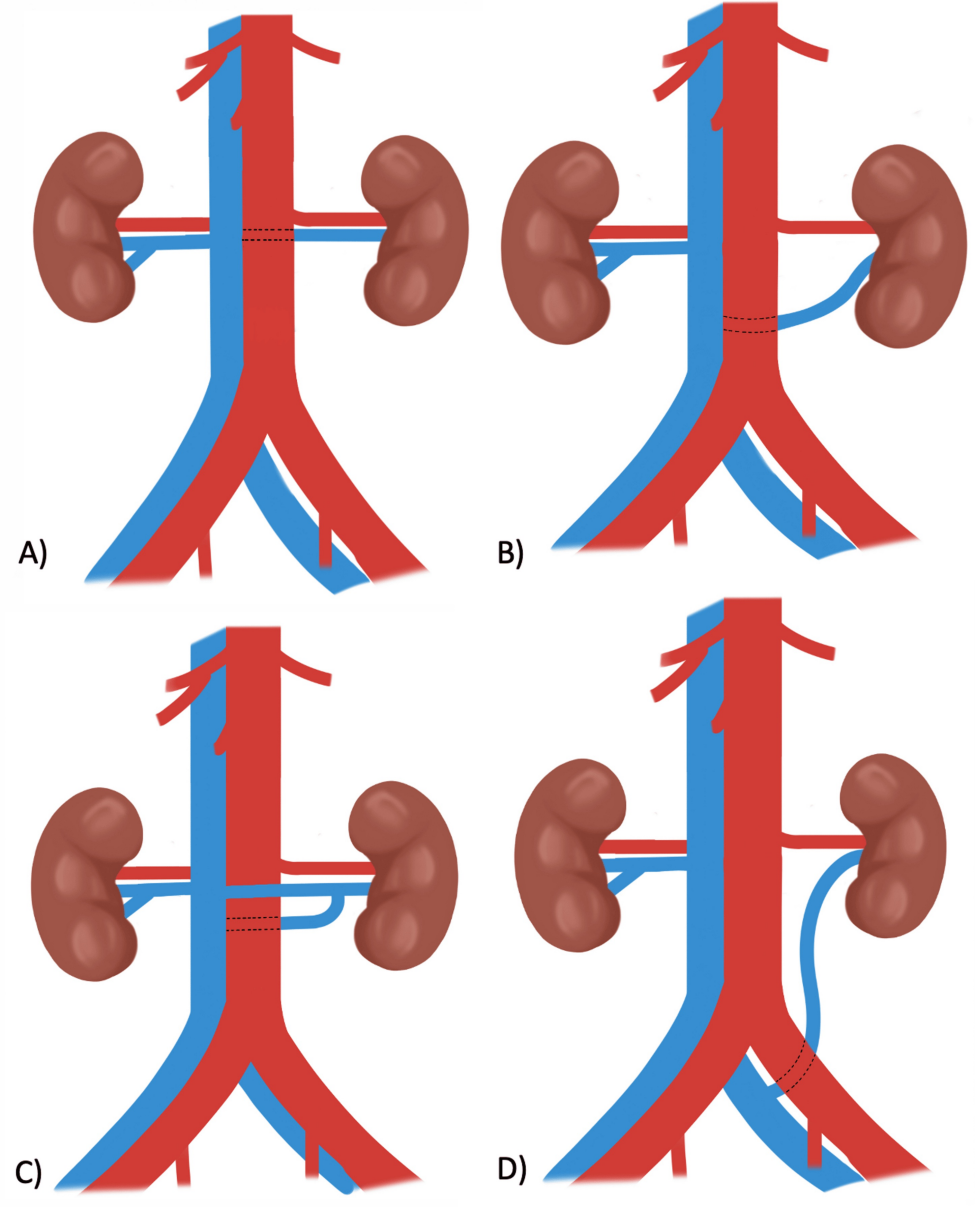
Schematic illustration of RLRV variants: (A) type I (orthotopic), (B) type II (caudal), (C) type III (circumaortic), and (D) type IV (iliac) RLRV: retroaortic left renal veins Digital illustration created by the authors

Congenital left renal vein anomalies are generally classified into four types based on their course and drainage point [8,9]: type I (orthotopic), the most common form, in which the vein passes posterior to the aorta and joins the IVC at the normal (orthotopic) level, typically between L1 and L2; type II (caudal), in which the vein drains into the IVC at a lower level, usually L4-L5, often receiving gonadal and ascending lumbar tributaries before confluence; type III (circumaortic), in which both preaortic and retroaortic limbs persist, forming a venous ring around the aorta (although technically distinct, it shares embryologic origins with RLRV); and type IV (iliac), the rarest type, which is characterized by an oblique, inferior course draining directly into the left common iliac vein, with only a few cases being reported (Figure 2).

In addition to these variations in venous trajectory, cases have also been reported describing trajectory variations occurring in combination with bifurcation of the retroaortic left renal vein.

The reported incidence of RLRV varies widely. Cadaveric and autopsy studies report a prevalence of 0.5-3.5% [2], whereas clinical studies utilizing advanced imaging, such as CT angiography, identify rates as high as 9.3% [2]. This discrepancy suggests that most RLRVs are asymptomatic and discovered incidentally during evaluation for unrelated conditions. A large meta-analysis, with more than 100 studies including both cadaveric and clinical studies, concluded in an overall prevalence of 0.030 (CI: 0.024-0.036) for RLRV (including types I, II, and IV) and an overall prevalence of 0.035 (CI: 0.028-0.044) for circumaortic left renal vein (type III) [10]. Despite its rarity, RLRV is clinically relevant in surgical contexts, occurring in approximately 0.8% of patients undergoing abdominal aortic aneurysm repair [11].

Although RLRV is typically asymptomatic, its retroaortic course predisposes it to posterior nutcracker syndrome (PNCS) [4]. PNCS results from mechanical compression of the RLRV between the abdominal aorta anteriorly and the vertebral column posteriorly [4], producing a significant pressure gradient and consequent left renal venous hypertension (LRVH). Symptoms of LRVH include flank or abdominal pain, hematuria (gross or microscopic), and proteinuria due to rupture of fragile renal fornices, particularly during exertion [12]. Collateral flow through the gonadal or ovarian veins may cause scrotal varicocele in men or pelvic congestion syndrome in women [13]. However, most RLRV cases remain asymptomatic; only about 6.6% of patients identified radiographically exhibit clinical PNCS [14].

Even asymptomatic RLRVs often operate under subclinical venous hypertension, relying on collateral drainage through the lumbar and gonadal veins [15]. In standard nephrectomy, early ligation of these peripheral collaterals is common to facilitate mobilization. In RLRV, however, excessive traction or premature ligation can sharply increase renal venous pressure, causing parenchymal bleeding or compromising nephron integrity during partial nephrectomy [16]. Since RLRV represents a static anatomical condition, while PNCS is a dynamic physiological process, preoperative planning may require functional imaging, such as Doppler ultrasonography, to assess venous compression and confirm hypertension [17]. In cases involving hilar renal masses, preoperative determination of PNCS status is essential to anticipate technical difficulty and intraoperative risk during vascular occlusion.

In minimally invasive radical nephrectomy, RLRV increases the risk of hemorrhage from avulsion or laceration of the retroaortic vessel. This risk is particularly high in type II or III variants, where the deep, fixed vessel may be mistaken for other retroperitoneal structures or injured during medial dissection [3]. Because hemostasis in this region is difficult, uncontrolled bleeding often necessitates emergent conversion to open surgery. Traditional nephrectomy techniques achieve hilar control late in the procedure; however, in RLRV cases, early vascular control, securing the vein deep to the aorta before kidney mobilization, is safer. The retroperitoneal approach offers more direct access to the posterior hilum and facilitates early ligation of the RLRV [18].

During nephron-sparing minimally invasive surgery, temporary atraumatic occlusion of the RLRV may be required during tumor excision and renorrhaphy. However, gaining adequate circumferential exposure for clamp placement, using bulldog clamps or rubber band tourniquets, is challenging due to the vessel’s deep, fixed position between the aorta and spine [1]. While gentle traction aids exposure, excessive traction risks avulsing the vessel from its junction with the IVC or iliac confluence, resulting in uncontrolled hemorrhage [1]. If PNCS is present, compression further restricts mobility, making safe clamp application hazardous. Preoperative evidence of severe compression or fragility may contraindicate venous clamping. In such cases, surgeons may proceed with arterial clamping alone, accepting higher intraoperative blood loss, or, if hemostasis is inadequate, abandon the nephron-sparing approach. Attempting to clamp a compromised RLRV risks thrombosis or rupture.

## Conclusions

The RLRV is an uncommon venous anomaly with important clinical and surgical implications. Although often discovered incidentally, its retroaortic course increases the risk of vascular injury during renal and retroperitoneal surgery, particularly in minimally invasive procedures. Understanding its embryologic basis and potential association with posterior nutcracker syndrome is essential for accurate diagnosis and management. Preoperative identification through contrast-enhanced imaging allows for appropriate surgical planning, early vascular control, and prevention of intraoperative complications. Awareness of this variant among clinicians and surgeons is critical to ensure safe operative outcomes and avoid preventable morbidity. Preoperative review of venous anatomy should be considered standard practice before any renal or retroperitoneal surgery.

## References

[1] Aichroth J, Fox T (2012). Retroaortic left renal vein. J Diagn Med Sonogr.

[2] Yi SQ, Ueno Y, Naito M, Ozaki N, Itoh M (2012). The three most common variations of the left renal vein: a review and meta-analysis. Surg Radiol Anat.

[3] Thomas TV (1970). Surgical implications of retroaortic left renal vein. Arch Surg.

[4] Skeik N, Gloviczki P, Macedo TA (2011). Posterior nutcracker syndrome. Vasc Endovascular Surg.

[5] Mathews R, Smith PA, Fishman EK, Marshall FF (1999). Anomalies of the inferior vena cava and renal veins: embryologic and surgical considerations. Urology.

[6] Kumar S, Neyaz Z, Gupta A (2010). The utility of 64 channel multidetector CT angiography for evaluating the renal vascular anatomy and possible variations: a pictorial essay. Korean J Radiol.

[7] Tanka M, Tuka F, Abazaj E (2018). Circumaortic right renal vein with multiple vascular anomalies. Radiol Case Rep.

[8] Karaman B, Koplay M, Ozturk E (2007). Retroaortic left renal vein: multidetector computed tomography angiography findings and its clinical importance. Acta Radiol.

[9] Shindo S, Kubota K, Kojima A (2000). Anomalies of inferior vena cava and left renal vein: risks in aortic surgery. Ann Vasc Surg.

[10] Hostiuc S, Rusu MC, Negoi I, Dorobanțu B, Grigoriu M (2019). Anatomical variants of renal veins: a meta-analysis of prevalence. Sci Rep.

[11] Drabe N, Zund G, Hoerstrup SP, Jockenhövel S, Vogt PR, Turina M (2001). Surgical management of retro-aortic left renal vein in combined abdominal aortic and coronary surgery. Vasa.

[12] Zhang H, Li M, Jin W, San P, Xu P, Pan S (2007). The left renal entrapment syndrome: diagnosis and treatment. Ann Vasc Surg.

[13] Shokeir AA, el-Diasty TA, Ghoneim MA (1994). The nutcracker syndrome: new methods of diagnosis and treatment. Br J Urol.

[14] Heidler S, Hruby S, Schwarz S, Sellner-Zwieauer Y, Hoeltl W, Albrecht W (2015). Prevalence and incidence of clinical symptoms of the retroaortic left renal vein. Urol Int.

[15] Gibo M, Onitsuka H (1998). Retroaortic left renal vein with renal vein hypertension causing hematuria. Clin Imaging.

[16] Heidbüchel H, Hennes B, Kaiser R, Buss H, Doppelfeld E, Kühnel W, Melchior H (1977). Renal function after ligation of the left renal vein. Eur Urol.

[17] Yang YR, Ling WW, Shi SH, Li YZ, Zhou JJ (2022). Ultrasound features of posterior nutcracker syndrome: case series and literature analysis. Quant Imaging Med Surg.

[18] Tatarano S, Enokida H, Yamada Y (2019). Anatomical variations of the left renal vein during laparoscopic donor nephrectomy. Transplant Proc.

